# Patterns of Cognitive Performance in Healthy Ageing in Northern Portugal: A Cross-Sectional Analysis

**DOI:** 10.1371/journal.pone.0024553

**Published:** 2011-09-08

**Authors:** Ana Cristina Paulo, Adriana Sampaio, Nadine Correia Santos, Patrício Soares Costa, Pedro Cunha, Joseph Zihl, João Cerqueira, Joana Almeida Palha, Nuno Sousa

**Affiliations:** 1 Life and Health Sciences Research Institute (ICVS), School of Health Sciences, University of Minho, Braga, Portugal; 2 ICVS/3B's, PT Government Associate Laboratory, Braga/Guimarães, Portugal; 3 Centro Hospital Alto Ave-EPE, Guimarães, Portugal; 4 Department of Psychology – Neuropsychology, University of Munich, Munich, Germany; Federal University of Rio de Janeiro, Brazil

## Abstract

**Background:**

The Minho Integrative Neuroscience Database (MIND)-Ageing project aims to identify predictors of healthy cognitive ageing, including socio-demographic factors. In this exploratory analysis we sought to establish baseline cohorts for longitudinal assessment of age-related changes in cognition.

**Methods:**

The population sample (472 individuals) was strictly a convenient one, but similar to the Portuguese population in the age profile. Participants older than 55 years of age were included if they did not present defined disabling pathologies or dementia. A standardized clinical interview was conducted to assess medical history and a battery of neuropsychological tests was administered to characterize global cognition (Mini Mental State Examination), memory and executive functions (Selective Reminding Test; Stroop Color and Word Test; and Block Design subtest of the Wechsler Adult Intelligence Scale). Cross-sectional analysis of the neuropsychological performance with individual characteristics such as age, gender, educational level and setting (retirement home, senior university, day care center or community), allowed the establishment of baseline clusters for subsequent longitudinal studies.

**Results:**

Based on different socio-demographic characteristics, four main clusters that group distinctive patterns of cognitive performance were identified. The type of institution where the elders were sampled from, together with the level of formal education, were the major hierarchal factors for individual distribution in the four clusters. Of notice, education seems to delay the cognitive decline that is associated with age in all clusters.

**Conclusions:**

Social-inclusion/engagement and education seem to have a protective effect on mental ageing, although this effect may not be effective in the eldest elders.

## Introduction

According to the United Nations Programme on Ageing, one out of every ten persons is now 60 years or above, with this number expected to increase to one out of five and one out of three by 2050 and 2150, respectively [1]. This is a noteworthy estimation when considering that ageing is associated with a gradual decline in cognition. Importantly, these numbers imply a responsibility for the society, including its researchers, to focus on identifying predictive factors of healthy mental ageing. Ultimately, the understanding of such elements will contribute to the development of strategies to reduce the health burden associated with mental ageing, as opposed to just a focus on the development of ‘treatments’ for the elder.

Research on normal ageing has shown that evolution of cognitive performance over the lifespan is not a uniform, but rather a heterogeneous process. On one hand, there is evidence of a significant negative relationship between age and cognitive abilities such as processing speed, explicit memory or verbal fluency [2]–[6]. On the other hand, performance in knowledge-based verbal abilities and verbal production, implicit memory and autobiographical memory are relatively spared until late in life [5]–[9]. Importantly, the presence of such distinctive patterns in the same participant has led some authors to propose that ageing has distinctive effects on the neural systems underling various cognitive, memory and discourse abilities [10]–[15].

Cognitive performance in healthy ageing is associated with a high inter-individual variability (diversity) as well as with a high intra-individual variability (dispersion). That is, even controlling for the effect of age, some elders perform better than others and the same individual may perform differently in different domains [10], [16]. Furthermore, diversity in cognitive performance has been associated with education [17]–[19], social engagement [20]–[22], economic resources [23] and genetic factors [10], [24], while dispersion has been associated with demographic, health and individual characteristics [16], [25]. Altogether, the influence of these multiple factors, including socio-cultural characteristics, on cognitive performance is complex; they do not affect homogeneously all cognitive domains and may in addition interact to yield unexpected or distinct cognitive responses [17], [26]. The elucidation of the differential effect of these factors on cognition in the elderly, and their interaction, is a crucial issue for understanding what determines healthy cognitive ageing, and why some elders undergo cognitive decline, which can lead to increased morbidity, functional impairment and disease risk [12], [27], while others do not. Individuals with successful coping with normal age-associated cognitive decline are assumed to have higher cognitive reserve, defined as active adaptation to this decline. Higher levels of intelligence (and thus cognitive capacities), and of education, occupational attainment and social engagement help to explain why some individuals demonstrate more efficient coping in older age than others since they have optimized (or even maximized) cognitive performance by more efficient use of brain networks or differential ability to recruit alternate brain network [28].

In this study we assessed performance in various cognitive domains in elders living in the Minho region of Northern Portugal. The registers were obtained from the Minho Integrative Neuroscience Database (MIND) database, which was created as a central repository of information on socio-cultural, cognitive, genetic, biochemical, neuro-structural and functional domains to establish and characterize baseline clusters, by cross-sectional analysis, for further longitudinal studies. Specifically, we were interested in clusters of socio-demographic and/or social-engagement characteristics that were related to distinctive patterns of neurocognitive performance.

## Methods

### Ethics statement

The study goals and tests were explained to all participants and all gave voluntary informed written consent. The study was conducted in accordance with the principles expressed in the Declaration of Helsinki. The study was approved by the national ethical committee (Comissão Nacional de Protecção de Dados) and by the local ethics review boards (Hospital Escola Braga, Braga; Centro Hospitalar do Alto Ave, Guimarães; and Unidade Local de Saúde do Alto Minho, Viana-do-Castelo/Ponte-de-Lima). All medical and/or research professionals involved in the study were asked to sign a responsibility document as specified by the national ethical committee.

### Population sample

The population sample under study was strictly a convenient one, but proportional to the Portuguese population in the age profile. It was composed of individuals older than 55 years of age, from both rural and urban areas, living in the Guimarães and Vizela areas of the Minho region in the North of Portugal. This sample is part of a larger sample of 4,000 individuals between the ages of 18 to 90 years. This cohort constitutes a convenient but representative sample of the population and comprises individuals listed in the Community & Family Health Centers of the two cities (a total of 183,146 citizens). The primary exclusion criteria includes participant choice to withdraw from the study, incapacity and/or inability to attend the evaluation assessment session(s) in the local health center, diagnosed dementia and/or inability to understand informed consent. The age and sex distribution of the database differ in less than 2% from the age and gender distribution for the Portuguese population estimated by the Portuguese authority on statistics, the Instituto Nacional de Estatística (INE) [29]. Northern Portugal is characterized by an ageing index of 103 (16% of the population, 65+ years old), very similar to the national index of 118 (21% of the population, 65+ years old), as reported in the last published population census published by the INE [29]. Concerning educational level, this population is primarily characterized by basic education (4 years) with low percent scores in secondary and higher education.

As a proxy to assess engagement in mentally stimulating activities, which was shown by Salthouse to attenuate age-related cognitive decline [27], the type of institution (setting) from where each participant was recruited was recorded. Individuals were either living/spending the day in day-care centers or retirement homes, or were still living in the community within their families or by themselves. Among elders still living in the community, some were enrolled in senior universities. These persons belong to the part of the population with higher economic resources and high autonomy and motivation, actively involved in daily or weekly sessions of educational and cognitive demanding experiences. In contrast, individuals living in retirement homes were characterized by lower economic resources, less active daily-environment, and few or no social/cognitive-demanding or challenging activities. Therefore, in order to obtain an almost ‘realistic’ representation of the aged population, the exclusion criteria were reasonably lenient; the only exclusion criteria considered for this study was age below 55 years, dementia and/or presence of disabling pathologies or disease, and inability to understand informed consent. A team of experienced clinicians performed a standardized clinical interview that addressed current medication and excluded disorders of the central nervous system (stroke, epilepsy and neurodegenerative disorders), as well as overt thyroid pathology. Supporting information (such as verification of current medication) on each individual was also obtained from nursing and/or care-taking staff at each institution. All individuals that met the inclusion criteria were asked to participate in the neurocognitive assessment.

### Neurocognitive tests

Neurocognitive/psychological tests were selected to accomplish a general characterization of the elderly's cognitive and functional profile namely with respect to memory and executive functions, the domains described as being more vulnerable to the effects of aging. Because knowledge-based verbal abilities and language production are characterized by a stable pattern in aging [6], we opted to only assess the language domain within a general screening test. The cognitive tests included the Mini–Mental State Examination (MMSE), the Stroop Color and Word Test (Stroop test), the Selective Reminding test (SRT) and the Block Design sub-test of the Wechsler Adult Intelligence Scale. A team of trained psychologists conducted the neurocognitive assessments.

The MMSE evaluation [30] is the most widely used cognitive mental status-screening test. It assesses orientation, word recall, attention and calculation, language and visuo-construction abilities. The number of correct items is used as variable. The MMSE has a maximum total score of 30, obtained by the sum of scores in the five sub-tests; a score of 20 – 24 suggests mild dementia, 13 – 20 suggests moderate dementia, and less than 12 indicates severe dementia [31]. Nonetheless, this threshold should be adjusted depending on factors such as education [31], [32]. In Portugal the elderly segment of the population tends to be much less educated when compared to other developed countries. Therefore, if education is not taken into account, Portuguese elder individuals may be wrongly categorized as cognitively-impaired because of fewer years of formal education. Traditionally a score of 23 is used as a standard in Portugal [33]; more specifically, a score of less or equal to 27, 22 or 15 indicates, respectively, cognitive deficit for individuals with 12 or more, 1 to 11, or 0 (illiteracy) years of school education.

The Stroop test [34] is based on the advantage of the ability to read words printed in colors more quickly than to name the colors of the printed words when the color of the printed word differs from the color the word actually names. This phenomenon is known as the Stroop interference effect and refers to the inhibition of an over-learned response by a competing response. The ability to resist interference is commonly used as indicator of selective attention, cognitive flexibility, and response inhibition, and is used as a tool to assess executive function [35]. The Stroop version we used consisted of 3 variations [36]. In the first condition (word reading; here termed “Words”, W), participants were asked to read words denoting different colors but printed in black. In the second condition (color naming; here termed “Colors”, C) participants named the color of a series of words printed in the denoted color. In the last condition (words in colors, also termed here “Word/Color”, WC) participants were asked to name the ink-color of a series of words that denoted a different color (for example, the correct response to the word “blue” printed in red color, is “red”). For each condition, “Words”, “Colors” and “Words in Colors”, subjects were asked to process as many items as possible within 45 seconds, and the number of correct items used as performance measures. Also, according to performance in these 3 conditions, it is possible to compute an interference score, measuring the extent of delay in naming incongruent “Words in Colors”, when compared to naming congruent “Words” or “Colors”: WC-[(WxC)/(W+C)].

The SRT [37] is a multiple trial verbal learning and memory task. A list of 12 words was read to the participants. In the first trial participants were asked to recall as many items as possible. Thereafter, in the subsequent trials, only the items that were not recalled in the preceding trial were read to the subjects. When a word was recalled on two consecutive trials, it was assumed to have entered long-term storage (LTS). Words recalled consistently in all subsequent trials were scored as consistent long-term retrieval (CLTR). The total number of recalled items in LTS and CLTR were used as performance variables.

Lastly, the Block Design sub test of the Wechsler Intelligence [38] (here referred as “Blocks”) was used to assesses perceptual organization and spatial problem solving. Participants were shown increasingly difficult patterns consisting of blocks with red, white, and red and white sides and are then asked to arrange the same pattern using blocks that have all white sides, all red sides, and red and white sides. The number of correctly arranged blocks was used as performance variable.

### Statistical analysis

The MMSE score threshold was determined for the sampled elder population, particularly given the recent findings showing that the operational cut-off values change over time [39]. To address this issue, a logistic regression, relating the proportion of errors (incorrect answers) with age, educational level and gender, was first used to better understand the relation between the MMSE and our particular population. To estimate the best MMSE threshold (cognitive impairment boundary) the procedure described in Grigoletto et al. [32] was utilized. In summary, the method involved three steps: (1) ranking (list) of the MMSE scores by age, (2) estimation of the median fifth percentile of the distribution and the median age for the 5% youngest and (3) consecutive dropping of the youngest individual and its replacement with the next youngest from the list generated in step 1. Steps (2) and (3) were repeated until the last individual was added. The analysis yielded a two-column vector: one with the median ages of each youngest group and a second with the MMSE scores representing the 5th percentile of the score distribution in that group. To simplify the relation between these two variables, a Poisson regression was used to relate the total number of scores with age. The model was further adjusted for gender and level of education. The best model was the one with the lowest Akaike Information Criterion (AIC) after applying a step-wise algorithm on the selection of the independent variables.

A multivariate regression tree analysis was used to build hierarchical groups of observations (T-clusters) and to select which explanatory variables (age, institutionalization setting, education and gender) affected the patterns of cognitive performance (as measured by the neurocognitive tests). This analysis constructs a hierarchical tree by repeatedly splitting the set of observations using a rule based on a single explanatory variable. At each split, the data are partitioned into two mutually exclusive groups; each of which is as homogeneous as possible. Splits are thus chosen to maximize the homogeneity (minimize the impurity) of the resulting two groups. Splitting continues until an overly large tree is obtained. Splits and clusters are characterized by the values and conditions of explicative variables. The terminal nodes are those that cannot be further split given that either a minimum number of observations or sufficient homogeneity has been reached [40].

Regression tree analysis was used as an exploratory method to gain information on the data and select the variables that better explain the patterns of cognitive performance of the elders studied. Following the method by De'Ath [40] the groups formed by the classification tree (T-clusters, which are based on a constrained cluster solution that is dependent on explicative variables) were compared with those obtained by an unconstrained cluster solution (C-clusters, which is known as Cluster analysis). The unconstrained solution was derived using K-means clustering around initial cluster centers given by the classification tree. When the results (clusters from each solution) were compared, if the unconstrained solution accounted for substantially more of the cognitive variation than the multivariate regression tree analysis, this could indicate that unobserved factors, additional to the explanatory variables in the tree, were responsible for the difference in the explained variability. On the other hand, if the two forms of analysis explained similar amounts of species variation, it is likely that important explanatory variables were identified. In such cases the membership almost coincides between the tree cluster groups and the K-means groups. Finally, clustering may be weak; in this case, if regression tree analysis can detect strong relations between explanatory variables and the multivariate patterns of cognitive performance, classification tree analysis may detect distinct groups not detectable by unconstrained K-means derived clustering. Clusters were described in relation to age, institution type and/or setting, education and gender.

A t-test was used to compare means with a Bonferroni correction when comparing more than two means and a chi-squared test or a fisher test to compare frequencies. A p-value lower than 5% was considered significant. Clusters were further analyzed to determine the relation between age and the scores on each test. The generalized linear model (GLM) with a data dependent link function was applied. The score distribution obtained from tests involving counts usually follows a Poisson distribution that can show overdispersion (that is the variance is higher than the mean). As such, a quasipoisson likelihood was used to estimate the parameters of the model when overdispersion was present. The dispersion parameter on each test was estimated as it gives information about inter-individual variability.

Although a study limitation, the urban vs. rural setting was not explored in the present analysis given the multiple underlying confounding factors that precluded a ‘clear-cut’ classification among the urban vs. rural groups. In fact, to accommodate all multiple divisions needed to characterize all area variables, the sample would have been too diluted (or the number of individuals in each group too small) for a significant comparison assessment.

Only individuals with all socio-demographic and neurocognitive measures (all tests completed) were included in the analysis. Missing values in the neurocognitive evaluations were mainly due to participant inability to read and/or write, as assessed during the administration of the test, particularly for individuals with no or very few years of school education. Furthermore, if individuals solicited for the evaluation session to stop, their choice was respected and the non-completed test invalidated if it could not be properly resumed and/or re-administered under the established guidelines.

The R statistical language [41] was used for the statistical analysis and for building accessory functions; namely, to follow the procedure described in Grigoletto et al. [32].

## Results

### Sample characterization

We interviewed 472 individuals with a mean age of 72.16 years (SD±8.11). Seventy four percent of the individuals were female and 26% male. Approximately 5% of the elders were illiterate, 60% had less than 4 years of formal education, 10% between 4 and 9 years and 25% more than 9 years of education. About one third (33%) lived in retirement (nursing) homes, 26% in day-care centers, and 41% in the community (in self or family homes, non-institutionalized). In the later group, 49% (representing 20% of the overall sample) were enrolled in senior university activities.

### MMSE threshold

To understand the MMSE test scores in this population a logistic regression model was first fitted relating the proportion of errors, obtained by each individual, with the variables age, education level and gender. All variables contributed significantly (p<0.001) to explain the proportion of errors in the MMSE. The estimated odds ratio showed that males made 20% less errors than females (OR = 0.79 IC 95% 0.71–0.89), independently of the educational level, and that elders with higher levels of education made 57% less errors than individuals with lower levels of education (OR = 0.43 IC 95% 0.38–0.49), independently of gender. Age increased the proportion of errors by 1% (OR = 1.03 IC 96% 1.03–1.04) for each year of ageing (that is, in 20 years the percentage of errors could increase by 20%).

**Figure 1:**
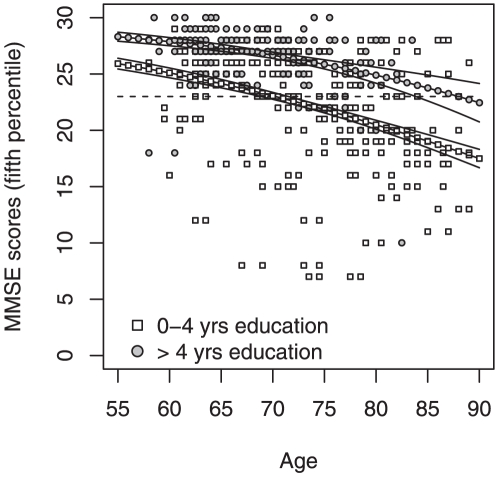
Mini Mental State Examination test scores. Fitted and observed values on cognitive decay for the fifth percentile of the MMSE scores as function of age and level of education.

Next, a Poisson regression model was used where the response variable was the fifth percentile of the MMSE scores by age. In this case, after adjusting for gender and level of education (up to 4 years of education or more than 4 years of education), the model with the lowest AIC had age and education as significant independent explanatory variables; furthermore, an interaction term between age and education was noted. This means that, although on average the total scores obtained in the MMSE test were different between males and females, this was no longer the case when considered the set of lower values representing the fifth percentile of the MMSE distribution by age. For the lower education level the fifth percentile was above 23 up to 71 years of age and then decreased, almost linearly, to 17 up to 90 years of age. For the higher education level the lowest MMSE score was always above 23 (Figure 1). From the analysis, a score of 23 points was used as the cognitive impairment threshold for elders with more than 4 years of education or up to 71 years old, and the fitted threshold values were used for those older than 71 with 4 or less years of education. After applying this rule 69 individuals were excluded from the original sample of 472 since their MMSE scores did not meet either criterion.

### Results of cognitive tests: identification of clusters

**Figure 2:**
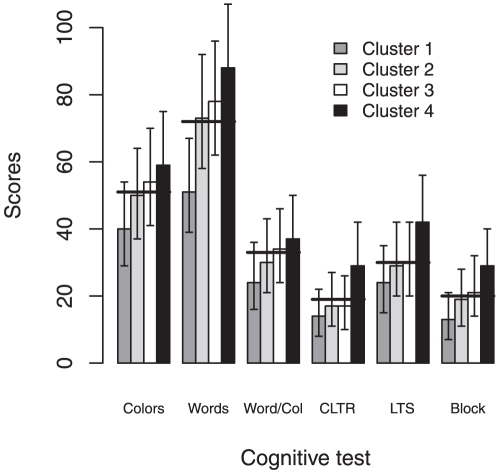
Neurocognitive assessments test scores. Overall (bold horizontal line) and cluster-specific (bars) geometric mean scores for each neurocognitive test. Clusters were identified by the multivariate regression tree analysis. The 95% confidence interval was estimated for the geometric mean for each cluster. Colors, Words and Word/Color indicate the Stroop conditions color naming, word reading and words in colors, respectively. Blocks designates the Block Design subtest of the Wechsler Intelligence. CLTR and LTS, both Selective Reminding Test assessments, refer to the total recalled items in the Consistent Long Term Retrieval and in the Long Term Storage, respectively.

In order to identify participants with similar characteristics, the best regression tree was selected by cross-validation within one standard error of the overall best. Based on this criterion, we found four clusters (terminal tree nodes) that were explained by institutionalization setting and education level (Table 1). Importantly, age and gender did not explain clustering; however, participants in clusters 3 and 4 were significantly younger than those in clusters 1 and 2 (Table 1). Hierarchically, institutionalization vs. non-institutionalization (setting) was the first most important variable to explain the variance associated with the cognitive profile. In fact, the four clusters could be grossly grouped in two categories. The first category, corresponding to clusters 1 and 2, comprised participants mostly sampled at nursing homes or day-care centers (i.e. institutionalized) whose performance was, on average, below the overall mean in each test. The second category, corresponding to clusters 3 and 4, comprised only non-institutionalized participants whose mean scores were above the overall mean (Figure 2). Education also contributed to the data disaggregation among the 4 clusters. The most extreme differences in cognitive performance were seen between institutionalized individuals with less than 4 years of education (cluster 1) and non-institutionalized participants with more than 9 years of education (cluster 4). Interestingly, performance of individuals from clusters 2 and 3 (institutionalized with 4 or more years of education, and non-institutionalized with less than 9 years of education, respectively) was similar in memory tests (CLTR and LTS) and perceptual organization/spatial problem solving (Block test) but not in executive functioning, as measured by the Stroop test, where cluster 2 participants showed poorer performance (Figure 2).

**Table 1:** Demographic and social characteristics of the clusters identified by the multivariate regression tree analysis (T-cluster).

	T-Cluster 1	T-Cluster 2	T-Cluster 3	T-Cluster 4	
Characteristics a	Number (%)				p-value
Type of social inclusion					<0.001
Retirement home	52 (42)	22 (63)	0	0	
Day-care center	72 (58)	13 (37)	0	0	
Senior university	0	0	30 (33)	58 (75)	
Other, in the community	0	0	61 (67)	19 (25)	
Education level					<0.001
Illiterate	2 (2)	0	0	0	
<4 years	122 (98)	0	63 (69)	0	
4 – 9 years	0	10 (29)	28 (31)	0	
>9 years	0	25 (71)	0	77 (100)	
Age (mean±sd)	73.6±8.4	73.4±8.2	69.3±7.1	67.6±6.1	<0.001^b^
Gender, female	95 (77)	25 (71)	60 (66)	60 (77)	0.229

a Only participants with no missing values in all variables were considered in the analysis (n = 327).

b The mean age is not different between clusters 1 and 2 and between clusters 3 and 4 after a t-test.

Overall, the institutionalization setting and education level could only explain about 31% of the variance associated with the patterns of cognitive performance. The first split based on the type of institution explained 21% of the cases, whereas education explained the remainder 10%. Therefore, K-means clustering was performed and the results from the two separate methods were compared (Table 2). Contrary to the tree clusters, K-means clustering with 4 clusters could explain 77% of the between-group variance. Membership between classic clustering (C-clusters) and clusters derived from the regression tree analysis (T-clusters) showed that C-cluster 1 had a higher proportion of elements from T-cluster 1 (72%) but the remainder was due to elements from T-clusters 2 and 3 (11% and 17% respectively). The members of this first C-cluster corresponded to elders with the lowest values in all scores. C-cluster 2, on the other hand, comprised members from all four tree clusters, but particularly from T-clusters 1 and 3, which represented 68% of the elders in the cluster. Furthermore, C-cluster 2 comprised individuals with an average value in all scaled variables near the mean value of 0. C-cluster 3 contained only a few members. Finally, C-cluster 4 represented 52% of elders from the T-cluster 4, and 26% from T-cluster 3, and comprised in addition also elders with positive scaled values for all cognitive variables. A chi-square inter-dependence test was performed between the two classifications, and a significant relation was found between the T-cluster and the C-cluster analysis (χ2(9) = 105.4; p<0.001).

**Table 2:** Cross-table with the number of elders (and row percentages) classified in the four clusters originated by the multivariate regression tree (T-cluster) and by the K-means clustering (C-cluster) analysis.

		Multivariate regression tree clustering			
		T-cluster 1	T-cluster 2	T-cluster 3	T-cluster 4
**K-means clustering**	**C-cluster 1**	65 (72)	10 (11)	15 (17)	0 (0)
	**C-cluster 2**	48 (33)	16 (11)	50 (35)	31 (21)
	**C-cluster 3**	2 (25)	0 (0)	4 (50)	2 (25)
	**C-cluster 4**	9 (11)	9 (11)	22 (26)	44 (52)

### Results of cognitive tests: relationship with age

**Figure 3:**
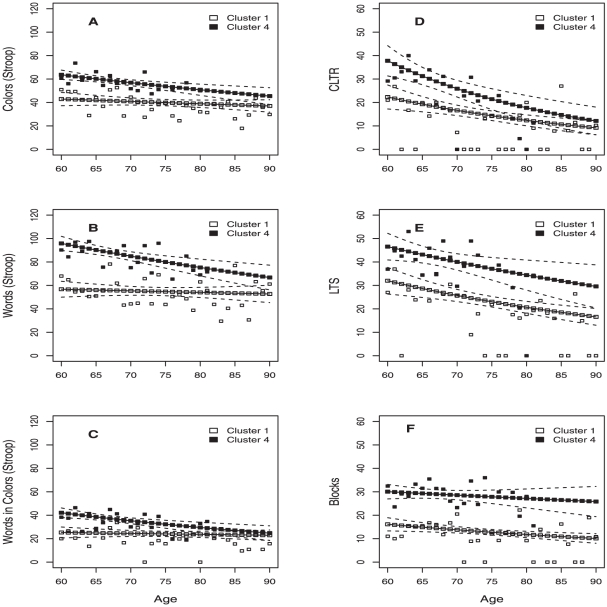
Fitted and observed values on cognitive decline for clusters 1 and 4. Fitted and observed values on cognitive decline, for each neurocognitive test, after adjusting a quasipoisson regression as a function of age and level of education. The 95% confidence interval was estimated for each of the fitted lines. Clusters were identified by the multivariate regression tree analysis.

Cognitive decline was modeled for T-clusters 1 and 4 only, given that they had the higher number of members coincident with the K-means clustering analysis (Figure 3). T-clusters 2 and 3 were omitted from the analysis since the latter had a small sample size and the former had its members distributed across all K-means clusters (possibly indicating that other factors besides education and type of institution are further discriminating factors). We found that the distribution of the scores in all neurocognitive tests had a variance much higher than the mean, suggesting that the distribution had a variance higher than expected for a Poisson distribution. Therefore, we used a quasipoisson regression model that took into account this overdispersion (Table 3).

**Table 3:** Parameters estimated from the quasipoisson model fit.

Cognitive test		T-cluster	Dispersion parameter	Age coefficient	p-value a
Stroop	Words	1	8.08	−2.6×10^−3^	0.06
		4	2.80	−0.01	<0.001
	Colors	1	6.83	−1.9×10^−3^	0.242
		4	1.88	−0.01	<0.001
	Words in Colors	1	7.90	−2.4×10^−3^	0.235
		4	2.93	−0.02	<0.001
Long Term Storage		1	8.07	−0.01	<0.001
		4	5.15	−0.03	<0.001
Consistent Long Term Retrieval		1	9.48	−0.03	<0.001
		4	7.79	−0.03	<0.001
Block Design		1	4.09	−0.01	<0.001
		4	2.42	−5.0×10^−3^	0.150

An age coefficient is shown for each T-cluster's cognitive test indicating if the scores on each test decrease or increase with age. Negative coefficients represent a decrease trend with age and positive coefficients a positive trend with age, as long as these coefficients are statistically significant.

a A p-value below 0.05 means that the trend slope was statistically significant.

Among T-cluster 4 individuals there was a significant age-related decrease in the scores for all neurocognitive tests, except for the block design test. For T-cluster 1 individuals, we found a significant decrease in performance in memory and block design performance with age (Figure 3 and Table 3). In addition, T-cluster 1 had a higher value for the dispersion parameter, estimated for each test, which was interpreted as a higher inter-individual variability when compared to T-cluster 4. Memory performance variables, in particular the CLTR, had the highest value for the dispersion parameter, independently of the group considered (Figure 3E), whereas the lowest value for the dispersion parameter was found for the model fit to the distribution of scores in the Block design subtest (Figure 3F). Specifically for the Stroop measures, after testing for pairwise correlation between the Stroop test variables (W, C and WC) a significant positive correlation between WC and interference was found with a value for the Pearson's product-moment correlation coefficient of 0.82 and a p-value lower than 0.001 (CI 95% 0.78–0.85). However, given that the number of participants with missing data in the variable interference is higher than in the variable WC we opted to use the later in the main analysis. Finally, the overall analysis indicated that inter-individual differences increased with age (Figure 3).

## Discussion

In its first stage, the project MIND-Ageing aims to describe and associate main factors (or cognitive reserve markers) involved in healthy cognitive ageing, such as years of education, social engagement and/or occupational attainment. The results herein described, for a sample of the population of a northern Portuguese region, show that non-institutionalization and higher levels of education are predictors of ‘healthier’ cognitive ageing. Importantly, and given that aging processes are complex and dynamic, this study constitutes a first step to cluster and further enlarge this sample for subsequent longitudinal analysis.

We found that a higher percentage of individuals with less than 4 years of education were below the MMSE cut-off of 23, particularly when compared to those with more than 4 years of education. This result is in line with other studies that used populations with a low educational background [42]. In addition, while a gender-dependent pattern of performance in the MMSE test was noted when the total number of scores was considered, this difference no longer existed when the fifth percentile scores (lowest score by age) were considered. This means that although male and females may score differently in the MMSE test, this may not hold true when the gender-score relation is calculated from the fifth percentile. In this regard, these observations differ from those reported for an Italian population where the curves representing the cut-off value where significantly different for males and females [32]. Nonetheless, it should be noted that while Grigoletto et al. [32] used groups ranging from 20 to 80 years of age, our population was comprised of elders between 55 and 90 years old, a difference that may account for the discrepancy of the results.

A limitation in our study is the small number of males particularly in the older age group. The number of males with lower levels of education and older than 70 years was 46, only 13 of them were over 80 years, and 6 over 85 years. In contrast, the female sample was composed of 54 individuals older than 80 years and 22 individuals over 85 years of age. It is of note that, in Portugal, the number of females with an age of 75+ years, and especially 85+ years, almost doubles that of males [29]. Nevertheless, altogether, our results clearly support the suggestion that normative rules for MMSE should be carefully assessed in populations with a low educational background [32], [42], [43]. Interestingly, however, the cut-off values that we adopted for elders with an age between 71 and 90 years (between 23 and 17) are consistent with the proposed cut-off scores in other studies in populations with a low educational background [32], [42].

With respect to patterns of age-associated cognitive performance, four different clusters emerged that were explained by the type of social inclusion (setting where the elders lived and/or spent the majority of their time in) and by the level of education. Specifically, our analysis split the cognitive scores in lower than average among elders sampled at retirement homes and day-care centers and higher than average among elders living in the community. The “use it or lose it” hypothesis of cognitive ageing predicts that engagement in physical, social, and intellectual activities in older adults can prevent the deterioration of cognitive abilities [27], [44]. The precise mechanisms underlying the protective effects of such activities on cognition in the elderly, and the cognitive domain that is most beneficial for this protection, remain unclear [44]. Physical activities, as well as control of cardiovascular risk factors, appear to have a positive effect on executive functioning as evidenced by a strong relation between performance in executive tests and physical activity [44]–[46]. Interestingly, the benefits of physical activity on memory performance are less obvious [46].

Our observations on the effect of social inclusion, as a proxy for an intellectually stimulating lifestyle [47], on cognitive performance parallel those of other authors. Motivation, lifestyle (including, physical activity and health), and a meaningful occupational and social environment, associated with higher cognitive demands, human interaction and communication, are associated with a reduced cognitive decline, suggesting a cognitive reserve potentiation [20]. In our study we did not use a specific measure for physical, intellectual or social engagement; rather, the setting of living was considered an overall indicator of social inclusion. In fact, on average, we found a higher level of activity and/or social and intellectual engagement in a senior university setting than in a retirement home. As we plan to further characterize these baseline cohorts in the ongoing longitudinal studies, we will thoroughly consider and evaluate their specific activity levels and relate these various forms of engagement to cognitive performance.

Besides social inclusion, the second most important variable in the formation of the four clusters was education. Our results indicate that education may also act as protective factor in elders. Independently from the setting, higher education level is paralleled by better performance in memory and executive measures in each group. These observations are in agreement with other studies suggesting that higher levels of education are associated with higher performance in cognitive measures, higher independence levels in healthy ageing [48], [49], and decreased risk of dementia [8], [17], [50]. Although education is the most studied factor that is beneficial for cognitive reserve [19], [51], occupational and leisure activities may also have a positive effect [51], [52]. Most importantly, these factors may act independently of each other or synergistically with one another [53]. In the present study, the outcome of the cluster analysis suggests lower social engagement and low education as possible risk factors for decreased cognition (as indicated by the extreme T-Cluster 1), while the opposite appears protective (T-cluster 4), yielding the worst and the best cognitive profiles. T-clusters 2 and 3 seem to represent a kind of transition between ‘poor’ and ‘good’ cognitive performers. This conclusion should be drawn with caution; T-cluster 3 is not homogeneous and almost all individuals could also be placed in one of the other clusters. Of note, T-cluster 3 mostly represents participants still living independently in the community, with individual, self-generated physical, cognitive, and social activities.

Interestingly, cognitive decline was also observed for subjects in T-cluster 4 in all cognitive domains tested, except spatial organization/spatial problem solving, despite higher scores in the same cognitive tests compared to other groups. In contrast, subjects from T-cluster 1 displayed only an age-related decline in memory abilities, while performance in executive function tests was poor in all ages studied. This observation was not unexpected, as previous studies have shown that the initial advantage of well-educated groups in their middle age is reduced later in life [17]. However, our data also showed that subjects with higher education can “gain” several years of good cognitive performance. Our results further indicate that the point of inflexion in executive functioning appears earlier in life when compared with memory functioning, but appears later in life in individuals in T-cluster 4. The age at which executive functioning peaks depends on the specific measure used [54], [55]. Executive functioning abilities peak in the early 20 s and show a flat plateau after 70 years of age, whereas verbal classification peaks in the 40 s, and verbal retrieval in the 50 s [55]. In this regard it is important to consider processing speed, for example, in the Stroop measures; it is possible that some elders would have performed at a higher level if they had more time.

Finally, it is known that inter-individual variability increases during the ageing process. The degree of variability seems to depend on the cognitive tests used. Ardila et al. [8] have found a large heterogeneity particularly in measures assessing executive functioning, while Christensen et al. [10] described high performance variability in memory tests. In the later study, differences were attributed to health, genetic APOEε4 and disability [10], [16]. Interestingly, we found that the cognitive measures with more severe decline with age, i.e. CLTR and LTS, are those with higher variability of scores in older ages. Finally, another novel finding in the present study is that higher education and social engagement were significantly correlated with a decrease in the variability of cognitive scores.

The main objective of the initial phase of the MIND-Ageing study was to explore the influence of socio-demographic factors on cognitive performance patterns in an aged population. By assessing cognitive variables in elders living in different social-inclusion settings (i.e. institutionalized vs. non-institutionalized), the analysis allowed the identification of baseline clusters for further in-depth longitudinal analysis. In this regard, various aspects should be further considered in ongoing studies including, in particular, the inclusion of more males in the study and a careful characterization of social and physical activities levels and of the area of residence (primary current, adult life previous to elder years and childhood). Nonetheless, so far, exploratory characterization of 472 elder individuals indicated that distinct patterns of decline and stability in cognition are foremost linked to social “engagement.” In addition, low educational level may have a cumulative effect on earlier cognitive decline. Furthermore, age-related cognitive functioning is a heterogeneous process, with some abilities declining earlier and more rapidly than others. Finally, although social-inclusion/engagement and education may have a protective effect on mental ageing, this effect may be effective only up to the eldest years. In the planned longitudinal studies, individuals representative of the extreme clusters will be fully characterized in magnetic resonance imaging studies and electroencephalogram parameters, as well as in biochemical and cardiovascular measures, and the results related to composite memory and executive function performance scores.
